# Synlabia after Severe Monilia Infections: A Case Report

**DOI:** 10.1155/2010/209021

**Published:** 2011-03-08

**Authors:** Samia M. A. Saied

**Affiliations:** Plastic Surgery Department, Sohag University, Sohag, Egypt

## Abstract

*Case*. A 25-year-old woman presented with acute urine retention with overflow 6 months after an inadequate treatment of severe monilia infections. Examination revealed complete adhesion between both labia majora. Division of adhesion was done with reconstruction by labial mucocutaneous flap. Complete recovery was achieved with good cosmetic outcome. *Conclusion*. Labial adhesions whatever their severity is can be surgically divided with complete correction by locally designed flap to reconstruct the introuitus with rapid recovery, good healing, and good cosmetic outcome.

## 1. Introduction

Labial adhesions are common in young girls and occasionally occur in elderly women [1]. It is hypothesized that the relative hypoestrogenic states of these age groups predispose them to labial adhesions [2]. Although surgical dissection is sometimes required, topical oestrogen creams and gentle massage usually lead to successful breakdown of adhesions in these groups within a few weeks [3]. Labial adhesions have also been described in reproductive age women secondary to female circumcision, lichen sclerosis, herpes simplex, diabetes, pemphigoid, and caustic vaginitis [3–6]. To our knowledge, monilial labial adhesions are rarely described in the medical literature.

## 2. Case Report

**Figure 1:**
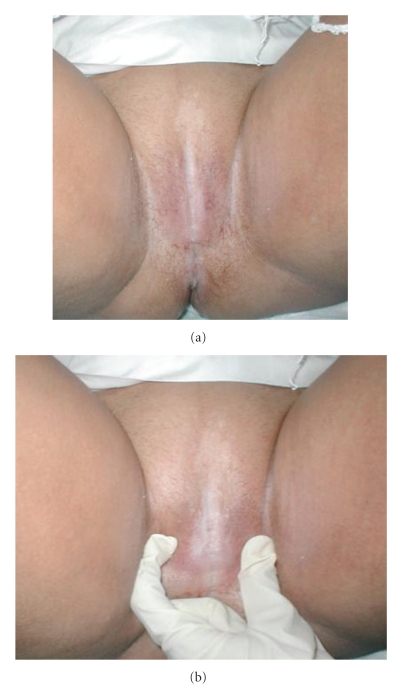
Preoperative photos showing adhesion with and without separation of vulva.

A 25-year-old woman, nulligravida, was divorced 5 years ago and was nondiabetic patient. Because of some Upper Egypt traditions she refused to go to doctors (as approximately all surgeons in our localities are male). She had a severe itching with severe monilia infection, and she consulted only dermatologic doctors, the conditions worsened till complete adhesion occured leading to urine retention with difficulty in micturition, then urine retention with overflow. On taking the history, she was circumcised as a routine procedure done for young female in villages and rural area in Upper Egypt. On examination she had a complete vulval adhesion with only pinhole opening for micturation with complete adhesion of the labia and overflow of the urine with some leucodermic area from chronic irritations (Figures 1(a) and 1(b)). She received antimonilia drug (Fluconazole).

Spinal anastasia with sedations was used. The area was infiltrated with injection of adrenalin in concentration of 1 : 10000. Separation of the adhesion and fusions was done, and bilateral vulvar flaps to reconstruct the defect of raw surface resulted. Randomized mucocutaneous flaps were dissected posteriorly to be advanced to reconstruct the raw surface after separation of adhesion. Urethral catheter was applied and lastly suturing of flaps using polyglactin (4/0) (Ethicon). She was instructed for standard perineal care, which included spreading the labia periodically and washing with water and antiseptic. The patient was discharged 48 hours after surgery with no complications and instructed to continue routine perineal hygiene at home.

**Figure 2:**
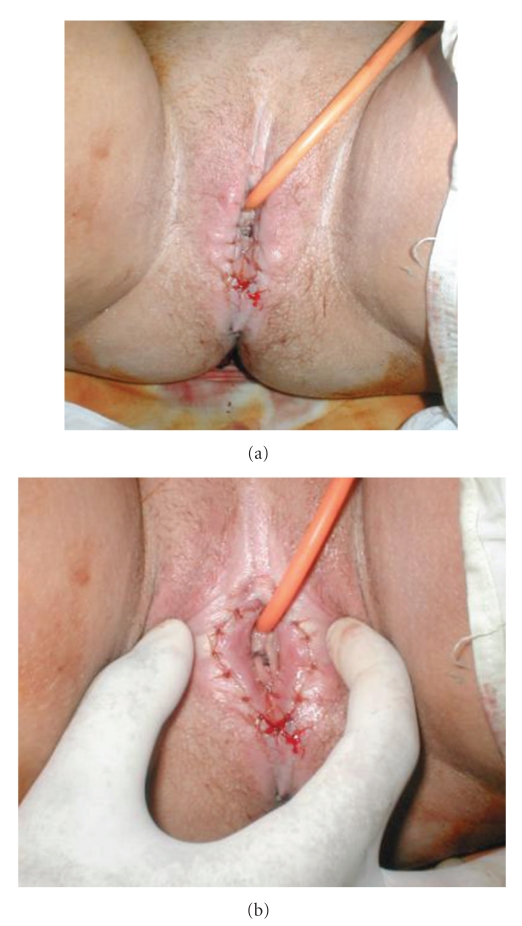
Postoperative photos showing separation of the of vulva using mucocutaneous flap.

Five days later she was seen in followup. She had resumed normal activity and was pain-free. Examination revealed that her labia were completely healed (see Figures 1(a), 1(b) and 2(a), 2(b)).

## 3. Discussions

Labial fusion is most commonly noted between 3 months and 4 years of age and has a peak incidence of 3.3% between 13 months and 23 months of age [7]. Labial fusion is considered to be an acquired condition secondary to hypoestrogenism and vulvovaginitis [8, 9]. Several previous authors hypothesized that the relative hypoestrogenic state of the immediate postpartum period contributes to the formation of labial adhesions [1, 10].

This middle age lady has no history of recent delivery and did not have any pattern of hypoestrogenic state. Severe and recurrent infection with bad hygiene can also predispose to labial adhesion, especially monilia infection. The local cultural problems led to the late presentation of the case till complete urine retention occured.

The fusion of distal based randomized mucocutaneous flap to reconstruct the defect has not been described in the literature before and the healing of the flap was excellent.
